# A qualitative study of the experiences of care and motivation for effective self-management among diabetic and hypertensive patients attending public sector primary health care services in South Africa

**DOI:** 10.1186/s12913-015-0969-y

**Published:** 2015-08-01

**Authors:** Katherine Murphy, Thandie Chuma, Catherine Mathews, Krisela Steyn, Naomi Levitt

**Affiliations:** Chronic Disease Initiative for Africa and Division of Diabetes and Endocrinology, Department of Medicine, University of Cape Town, Cape Town, South Africa; Department of Public Health and Family Medicine, University of Cape Town, Cape Town, South Africa; Chronic Diseases Initiative for Africa, Department of Medicine, Groote Schuur Hospital, Observatory, J 47-85 Old Main Building, 792 5 Cape Town, South Africa

**Keywords:** Diabetes, Hypertension, Self-management, Lifestyle modification, Motivation, Self determination theory

## Abstract

**Background:**

Diabetes and hypertension constitute a significant and growing burden of disease in South Africa. Presently, few patients are achieving adequate levels of control. In an effort to improve outcomes, the Department of Health is proposing a shift to a patient-centred model of chronic care, which empowers patients to play an active role in self-management by enhancing their knowledge, motivation and skills. The aim of this study was to explore patients’ current experiences of chronic care, as well as their motivation and capacity for self-management and lifestyle change.

**Methods:**

The study involved 22 individual, qualitative interviews with a purposive sample of hypertensive and diabetic patients attending three public sector community health centres in Cape Town. Participants were a mix of Xhosa and Afrikaans speaking patients and were of low socio-economic status.

**Results:**

The concepts of relatedness, competency and autonomy from Self Determination Theory proved valuable in exploring patients’ perspectives on what a patient-centred model of care may mean and what they needed from their healthcare providers. Overall, the findings of this study indicate that patients experience multiple impediments to effective self-management and behaviour change, including poor health literacy, a lack of self-efficacy and perceived social support. With some exceptions, the majority of patients reported not having received adequate information; counselling or autonomy support from their healthcare providers. Their experiences suggests that the current approach to chronic care largely fails to meet patients’ motivation needs, leaving many of them feeling anxious about their state of health and frustrated with the quality of their care.

**Conclusions:**

In accordance with other similar studies, most of the hypertensive and diabetic patients interviewed for this study were found to be ill equipped to play an active and empowered role in self-care. It was clear that patients desire greater assistance and support from their healthcare providers. In order to enable healthcare providers in South Africa to adopt a more patient-centred approach and to better assist and motivate patients to become effective partners in their care, training, resources and tools are needed. In addition, providers need to be supported by policy and organisational change.

## Background

Both diabetes and hypertension contribute significantly to the burden of non-communicable disease (NCD) in South Africa (SA). The prevalence of diabetes is estimated to be 9.0 % in the age group 30 and older (approximately 2 million cases) [1] and in a recent population based survey, 78 % of South Africans over 50 years of age were found to have hypertension [2].

The responsibility for the management of the majority of patients with NCD and/or their risk factors falls on the public sector, primary health care services, which are delivered at a community and district level and are mainly nurse driven. Approximately 17 million visits to Department of Health clinics per annum are related to hypertension and diabetes [3]. However, the early diagnosis, management and outcomes of care for patients attending these services are sub-optimal and few patients are achieving adequate levels of control; despite attending facilities on a monthly basis [4]. For example, a large study conducted in 18 primary care clinics showed that 67 % of patients with hypertension had BP >140/90 mm Hg and 58 % of patients with diabetes had non-fasting blood sugar levels >11.1 moll/L [5].

The national Department of Health has acknowledged that there is a pressing need to improve the quality of NCD care through appropriate training, the provision of resources and the reorganisation of primary care so as to accommodate a more concerted focus on health promotion and disease prevention [3]. In line with WHO recommendations, they propose a shift to a more patient-centred model of chronic care, which emphasises the importance of empowering patients to play an active role in self-management by enhancing their knowledge, motivation and skills for behaviour change, as well as their self-efficacy to carry out the behaviours necessary for long term self-care in their life context.

However, there is a paucity of data in South Africa on NCD patients’ current experiences of chronic care, their perspectives on and their capacity for self-management. Additionally, there is little understanding of what they might need from healthcare providers that would empower them to become active partners in their long term care. The aim of this study was to explore these issues among people with diabetes and hypertension attending public sector health-care services Cape Town, to contribute to a greater understanding of what patient-centred models of care may mean from the patient’s perspective and how interventions can be tailored accordingly, to take into account their needs and preferences.

**Fig. 1:**
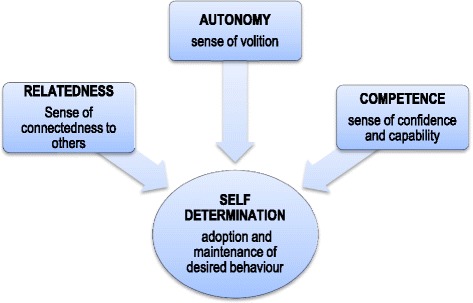
Concepts from self determination theory

Self Determination Theory (SDT) underpinned the study [6] as it provides a useful framework for exploring patients’ capacity and motivation for self-care and the extent to which the healthcare system serves to either enhance or impede this. Over the past 15 years a growing body of research has confirmed the utility and efficacy of the SDT model in behaviour change interventions for tobacco dependence, diet, physical activity, glycaemic control, medical adherence and dental care [6] (Fig. 1).

According to this theory, patients are more likely to identify with and internalise the need to adopt healthy lifestyle behaviours or adhere to treatment when their basic psychological needs for autonomy, competence and relatedness are supported. Patients feel *autonomous* when they regulate their behaviour volitionally, rather than because of external pressure or coercion; feel *competent* when they are informed, capable and effective in achieving desired outcomes and feel a sense of *relatedness* or connection with others when they feel respected, understood and cared for. Importantly, gaining a sense of competency is facilitated by autonomy - that is once a person is volitionally engaged and has personally endorsed or identified with the value or importance of a behaviour change, they are then more likely to learn and apply new strategies and skills to initiate and maintain change, and ultimately achieve better health outcomes. In contra-distinction to self-efficacy theory [7], SDT predicts that competence alone is not sufficient to ensure adherence; it must be accompanied by autonomy. In the context of NCD care, there are several ways in which clinicians can assist patients develop competency and support autonomy: they can elicit and acknowledge the patient’s perspective; offer choices about treatment options; prompt a meaningful rationale for lifestyle change from the patient themselves, and support the patient in an exploration of how to change, without applying pressure that detracts from a sense of agency and choice. In this sense, SDT is highly congruent with the philosophical tenets of patient-centred care and more particularly, with the behaviour change counselling methods of motivational interviewing [8].

## Methods

We conducted a qualitative study comprising individual, in-depth interviews with hypertensive and diabetic patients. The research was undertaken in 3 out of the 44 public sector Community Health Centres in the Cape Town metropolitan area that provide free, primary healthcare services for those unable to afford private health insurance. The clinics were selected under the direction of the Western Cape Provincial Department of Health as typical of public health services and situated in residential areas of low socio-economic status.

A criterion sampling procedure was used to select patients who had diabetes and/or hypertension; were over 18 years old; had received a diagnosis of hypertension or diabetes within the last 10 years, but for longer than the past 3 months; who were willing to be interviewed within the clinic environment; able to communicate in simple English and provide informed consent. Patients were approached while they waited in the queue for their appointment, questioned briefly to determine whether they fitted the selection criteria and then asked if they would agree to an interview, either while they continued to wait for their appointment or soon afterwards. The interview took place in a private venue on the clinic premises.

The interviews were conducted in simple English, but an interpreter who could speak both the local vernaculars, Xhosa^1^ and Afrikaans^2^, was on hand to assist participants who needed to explain something in their mother tongue. This was then interpreted back into English for the benefit of the interviewer, who was not proficient in these languages. This procedure was explained during the recruitment process.

The format of individual qualitative interviews allowed for the in-depth and confidential exploration of participants’ experiences, opinions, beliefs, feelings and actions. An interview schedule was used to ensure that key topics relating to study objectives were covered during the discussion. The schedule included questions which aimed to elicit patients’ personal experiences of chronic disease care; their perceived competence, autonomy and motivation for self-management, as well as perceived barriers. The questions were broadly framed and open-ended and the structure of the interview was flexible in order to give participants the opportunity to shape their own narratives and raise issues of importance to them. For example, one of the open questions used to elicit patients’ experiences of care was: “Please can you tell me about your experience of the visit when the doctor told you that you had diabetes/hypertension….”

The strategy of *member checking* [9] was used during the interviews to minimise researcher bias. This involves restating and summarising key information provided by the respondent by use of reflective summaries to test the accuracy of the researcher’s understanding.

A brief questionnaire was administered prior to each individual interview, which collected basic demographic information. Both research instruments were piloted before fieldwork commenced.

Data analysis of the verbatim, transcribed interview texts proceeded according to the discrete steps for qualitative content analysis as outlined by Graneheim and Lundman [10] and Elo and Kyngas [11]. After an initial reading of all the available transcripts, ‘units of meaning’ in sentences or paragraphs were described in the abbreviated form of a code. The codes were then grouped into more abstract categories with various dimensions or characteristics and finally, these were organised into the core categories or themes guiding the investigation, which were derived from Self Determination Theory. Thus analysis was both a deductive and inductive process in that the data was scrutinised for a priori issues relating to the research questions, but also captured unanticipated explanations and issues raised by the respondents. An analytical framework was ‘workshopped’ among members of the research team before being applied across all the interviews as a means of grouping views, experiences and quotes according to their appropriate thematic reference.

Once the first ten interviews were completed, data analysis started and then proceeded contemporaneously with further sampling. Once no new information was being obtained for on-going thematic development, sampling ceased. This point was reached after 22 interviews. Ethical clearance was obtained from the University of Cape Town’s Human Research Ethics Committee. Written consent was obtained from all participants after an explanation of the study was provided.

## Results

### Demographics

Of the 22 respondents interviewed, nine had hypertension, six had diabetes and seven had both. Twelve respondents reported various other co-morbidities, which included: HIV, gout, breast cancer and arthritis. All participants were taking prescribed medication for their condition.

More females were willing to be interviewed than males. As a result, 16 women were interviewed and only six males. The age range was between 30 and 75 years; 12 spoke Xhosa^3^ as their first language and ten Afrikaans.^4^ In terms of education, six reported having only had a primary school education; 11 reported that they had a high school education and the remaining five participants had some form of tertiary education. Fourteen considered themselves employed, but only four had formal employment. Sixteen were either single or widowed and 6 were currently married.

A minority of respondents - 4 out of 22 - were found to have had successfully initiated and maintained behaviour change in response to their diagnosis. These four had significantly modified their diet over time and two were physically active on a regular basis.

### Themes

The findings are presented in accordance with the key variables theorised to determine motivation for health-related behaviour change in the SDT, namely: *relatedness, autonomy support* and *competence.*

### Theme 1: Relatedness with healthcare providers

This theme refers to the extent that patients felt understood, cared for and valued by their healthcare providers. According to SDT, a lack of relatedness can influence a patient’s process of coming to terms with their chronic condition and impact on the level and quality of their motivation to control their condition through lifestyle modification and adherence to medication [6]. Patient’s descriptions of their experiences pointed to a number of issues which suggested that providers had failed to establish a sense of relatedness with them. These included: providers not having the time, communication skills or inclination to explain the cause and nature of the illness to patients or to counsel them on self-management; a lack of emotional support and reassurance for patients, particularly at the time of diagnosis, and accounts of providers alienating patients by being impatient and rude.

For example, *When I came to the clinic, tests were done and the doctor just recorded a lot of things in the folder. They didn’t really explain anything to me, so even today; I really don’t know why I have this thing or what to do. I am worried (Female, 65 years old).*

The failure to offer an adequate explanation at diagnosis, of the aetiology and nature of the illness left many patients feeling shocked and confused and in need of emotional support. Twelve out of the 22 participants reported feeling high levels of anxiety and stress when they received the diagnosis, because they believed it to be a certain ‘death sentence’ – a misconception which healthcare providers failed to identify and dispel. For example, *When the doctor told me that was diabetic, I thought that I was going to die or have to cut my legs off. I was so scared! (Female, 31 years old).* This patient had not divulged her fears to the doctor and had remained unaware for some time that it was possible to effectively manage the condition to avoid complications.

The consequence for another patient, who believed that diabetes could be cured after several months of treatment, was a lingering distrust of the doctor and the diagnosis: *I believe the doctor was lying to me when she said it’s just a normal thing, because a normal thing reaches a point when it gets out of the body. But I find that its three months now and I am carrying on and on… So far, I am taking my medication, but after 4 months (if I am not cured) I will drink Aloe Vera if necessary (Male, 34 years old).*

The desire to receive more information and counselling from healthcare providers was a strong emergent theme. Patients specifically wanted healthcare providers to spend more time explaining why and how they had come to have diabetes or hypertension and to provide practical guidance on self-management and lifestyle modification. However, they were uncertain about asserting these needs, either because of a lack of confidence in asking this of an authority figure, or in some instances, out of consideration for the time pressures faced by providers in the public sector: *I know the doctors are under pressure and there’s the next patient waiting, but I just wish that they could be more detailed, sort of give us more information, talk with us about what this whole thing entails (Female, 46 years old).*

Several other patients described how the negative attitudes of healthcare providers inhibited their ability to be open with and trusting of them. This finding was common across all three clinics. Patients complained about nurses in particular, being impatient, abrupt and disrespectful. For example: *When it comes to the nurses it is really bad. When you go inside to see the doctor, he will talk to you fine, but when it comes to the nurses, hm-mm, they are so rude. It’s not a good experience (Female, 50 years old).*

Another patient recounted being frequently reproached by doctors for not successfully controlling his glucose levels. He interpreted this as the doctors having little empathy for his struggles and therefore felt unable to explain his difficulties to them or to ask questions about how else he could improve his level of control: *The doctors always complain because every time they check my sugar it is very high. I just don’t understand, because I have cut out all the sugar, no cakes, sweets or anything of that sort (Male, 75 years old).*

These negative experiences affected patients’ ability to accept their condition, as well as their motivation to engage with providers around lifestyle issues and other concerns.

In contrast, there were four respondents who described their interactions with healthcare providers in positive terms, expressing an appreciation for being given information and practical advice: *I remember the doctor told me about what to eat, what not to eat. It was like, some sort of a counselling. And I was given a paper on the right diet for high blood pressure…. (Female, 34 years old).*

*The sisters sometimes tell us things like, what to wear on your feet and that shoes shouldn’t be too tight. So, they’re teaching things that we didn’t know (Female, 62 years old).*

### Theme 2: Experiences of autonomy support

This theme relates to the extent to which the healthcare provider empowers the patient with a sense of control and choice over health related decisions. None of the patients described an experience of a healthcare provider actively encouraging their collaboration or fostering power-sharing in the consultation. The overall impression was that the traditional model of provider-patient interaction prevailed, where the provider assumes a dominant, expert role and determines the agenda of the consultation, leaving the patient with little or no opportunity for interaction.

It appeared that with a few exceptions (4), patients did not have the necessary knowledge to enable them to actively engage in decision making about treatment, self-management or lifestyle modification. *I am not sure what it (diabetes) is, but at least what I am doing is just to always get my medication (Female, 58 years old).*

Furthermore, it appeared from some patient accounts that when healthcare providers did proffer relevant information, they were not encouraged to express any response or opinion. This suggests that providers did not elicit their perspectives or act to inspire feelings of control or choice, for example: *I was told I must eat differently, but the doctor never discussed with me how I must eat (Male, 34 years old).*

As a result of a lack of engagement with these issues, some patients had not yet personally endorsed or identified with the value or importance of self-management and lifestyle change, but reported merely complying with the medical treatment as prescribed: *I just follow instructions as the doctor said to me. I decided ok, the doctor told me this and this, which is why I have to follow it, because they know what I don’t know. They saw it in my body…so I just carry on.. (Male, 34 years old).*

Some patients also reported having had questions, but feeling apprehensive about asking them. For example, one of the respondents explained that he was very unhappy with his medication, but did not raise the issue with the doctor, because he expected to be reprimanded for not having succeeded in lowering his blood pressure. In this case, the patient’s inability to resolve his concerns about medication had implications for his adherence to treatment: *I am not happy. I feel dizzy and tired…… I think that this is the wrong medication. I wish that the doctor can change that tablet that I am using… Even today, I was telling myself to ask the doctor if she can change my medication, but I am worried about what she will say to me….. (Male, 34 years old).*

These quotes illustrate how a lack of understanding, coupled with a lack of autonomy support, limited the ability of some patients to develop the kind of intrinsic motivation hypothesised by SDT to be important for long term self-care.

### Theme 3: Competency for self-management

A striking finding across the interviews was the low level of health literacy among patients (defined as the extent which an individual can obtain, process and understand the basic information and services needed to make appropriate decisions regarding their health) [12].

Only about half the sample reported having received some form of information at the clinics, which suggests that the provision of health education for people with hypertension and diabetes attending these facilities is, at best, unsystematic or inconsistent. Nine patients recalled receiving brief, prescriptive advice from doctors or nurses on some aspects of self-care; ten had received some form of written material (either a small pamphlet or diet sheet) and one patient had received a referral to a dietician from their doctor. A general lack of education materials was verified by the researcher’s own observations: the only evident materials at all 3 clinics were some posters containing messages on the symptoms of diabetes, foot care and dietary advice.

Without adequate information from healthcare providers and without an attempt on their part to understand patients’ existing levels of knowledge or beliefs about their illness, problematic misconceptions about the causes of Type 2 diabetes and hypertension prevailed. In addition, only a few patients (4) appeared to understand the role of lifestyle in the development and control of these conditions. This often resulted in a lack of agency, with consequent negative implications for self-care.

For example, two respondents believed their hypertension to be caused by an external agent contaminating their blood: *I don’t know the cause of this thing or how it got into my blood. My father told me my high blood pressure could be a sign that my blood is dirty, but I don’t know (Male, 34 years old).*

Another common belief was that hypertension was *a disease of the nerves*, caused by emotional upset: *I have high blood pressure because my son was shot in 2006. I never went for counseling then, so afterwards it worked on me. That’s why I have high blood pressure (Female, 54 years old).*

In two other instances, a belief that their hypertension was inherited and that there was nothing one could have done to avoid it, resulted in patients fatalistically resigning to the condition: *My grandmother used to have high blood pressure. So the day that I was diagnosed, I was waiting for it (Female, 35 years old).* Other patients, on the basis of certain prevailing lay beliefs, misdirected their efforts in seeking effective treatment. For example, several patients reported using alternative remedies such as Aloe Vera or ‘holy water’, or attempting to ‘clean their blood’ to cure their illness: *There is a lady who told me that this (diabetes) is caused by dirty blood. She told me that I must clean my blood with a Vitamin B injection from the chemist and then my blood will be okay (Female, 62 years old).*

In two cases, patients mistakenly believed that the symptoms of other health problems were associated with their chronic condition and had failed to seek appropriate treatment for them: *Ever since I got this high blood pressure, when I go to the toilet my urine feels very hot. And sometimes when I am having sex it burns. I don’t know if it’s because of this thing in my body (high blood pressure). I am really not happy with this (Male, 34 years old).*

Most patients (18) expressed a lack of confidence in undertaking self-care and an uncertainty about how to go about modifying their lifestyle. Lacking in the necessary competency, they looked to healthcare providers for input and assistance and were disappointed when this was not forthcoming: *They (the doctors) didn’t tell me anything (about my lifestyle). They just did a blood test and gave me medication….. that is all (Female, 34 years old).*

In some instances, it was clear that patients were willing to make lifestyle changes, but needed more detailed information from their healthcare provider in order to be able to implement these: *The doctor said that I must leave (out) the salt in the food because the danger is the salt. But she didn’t explain what kind of food I must eat. So, I don’t know what I must eat at home…(Male, 34 years old).* In other instances, patients had taken certain steps to modify their lifestyles, but described various barriers, such as a lack of support at home or the cost of healthier foods, which limited their ability to initiate or maintain behaviour change (these aspects are to be reported elsewhere). Many also expressed anxiety about their current state of health and complained of various symptoms, which suggested that they were not well controlled.

In contrast, there were four patients who demonstrated relatively good knowledge about the role of lifestyle modification in managing their condition, reporting that they had actively sought out information from the mass media: *From the day hospital, I went onto the internet, read books, um, talked to other people. I found out a lot of the things……(Female, 46 years old).*

In addition, these respondents expressed confidence in their ability to make appropriate decisions and to cope with their condition, and reported having implemented and sustained lifestyle changes: *Exercise became very important to me after I was diagnosed with high blood pressure. I started going to gym. I am also watching my diet now (Female, 46 years old).*

*I am part of an exercise group. I started last year and now I go every Thursday. I am really happy about joining. We are about 15 and everyone has a chronic illness, so we share ideas (Female, 66 years old).* For this patient, the assistance she had received from her providers had been a direct catalyst for behaviour change*: The doctor gave me information about diet and exercise and the sister referred me to the club… that is how I joined.*

## Discussion

Structured, patient-centred, self-management education and behaviour change counselling has been recommended as the cornerstone of effective diabetes and hypertension management in numerous guidelines [13]. Such an approach aims to enhance *patient activation* (the patient’s willingness and ability to participate in decision making and take independent action to manage their health in their life context) through the building of a collaborative and autonomy supportive relationship. There is a growing body of evidence linking patient activation to the adoption of healthy behaviours, improved clinical indicators (including reductions in HbA1C, cholesterol and blood pressure), more positive experiences of care and lower healthcare costs [14, 15].

The fact that most patients in our study reported not having received adequate information; counselling or social support from their healthcare providers put them at a significant disadvantage in developing the *activation* necessary to manage their condition. An awareness of this deficit, accounted for the anxiety and frustration that many patients expressed about their state of health and quality of care. It is possible that this level of support from healthcare providers is perhaps even more important for patients of low socio-economic status who, as with our sample, often lack the health literacy, material resources and self-efficacy to cope with the complex burden of self-care.

Other South African studies have reported similar findings among NCD patients attending public sector health services. In a study by Mshunqane et al. [16], patients displayed a significant lack of knowledge on the role of lifestyle modification in preventing diabetes complications and were confused about the chronic nature of the disease. Three other qualitative studies among patients in rural areas of the North West, Western Cape and Limpopo provinces have also reported a critical lack of knowledge about the causes of Type 2 diabetes and hypertension, with consequent negative implications for adherence and control [17–19].

The problems with *relatedness* described by the participants in this study have also been reported by chronic patients in the rural areas of the Western Cape [18] and Gauteng Provinces [20]. In the latter study, the lack of relatedness was found to be among the reasons why some hypertension patients preferred to consult with traditional healers. Respondents perceived them as having more time for interaction, as well as being be more knowledgeable, understanding and accommodating of patients’ wishes regarding their treatment compared to ‘Western trained’ clinicians. Goudge et al. [21] also reported that poor interaction with providers led to some patients giving up on the public health system and going ‘healer shopping’.

Such characterisations of patient-provider interactions are not unique to SA. For example, Heisler et al. [22], who interviewed African-American and Latino diabetics in Detroit, found patients had joined a community-based programme out of frustration with a lack of information from healthcare providers and their focus on medication, at the expense of lifestyle modification. These patients reported feeling more comfortable discussing their concerns about self-management with community health workers. Fort et al. [23], mention the “vertical style of communication” with providers as being a significant barrier to effective self-management among diabetic and hypertensive patients in Mexico and Costa Rica. And Mathew et al. [24] in another qualitative study in Toronto, Canada, highlight older diabetic patients’ expressed needs for support from their doctors – for both the affective and practical aspects of self-management.

However, from the perspective of healthcare providers, both in SA and elsewhere, there are a number of common barriers to the provision of NCD care that would qualify as patient-centred and empowering. These include a lack of: time for the provision of education and counselling; training in appropriate communication skills; patient educational resources and aids, as well as a lack of continuity in care [25, 26].

Training has been shown to be effective in overcoming some of these barriers as it can demonstrably teach communication skills, change negative attitudes about the delivery of lifestyle interventions, as well as the style of interaction in behaviour change consultations so that they are more autonomy supportive [8, 26]. However, clinician training has very limited impact on practice if not accompanied by health system reform which supports a greater emphasis on health promotion and disease prevention as a standard of good care; promotes a consistent approach across the entire chronic care team; allows more clinician time for education and counselling and develops effective referral linkages with allied services and community-based resources [27, 28]. To this end, research is urgently needed in our setting to investigate what kinds of organizational changes are required to accommodate patient-centred models of chronic care and how to upscale them. The proposed integration of NCD/HIV chronic care may facilitate the transfer of lessons from the considerable success of local antiretroviral therapy (ART) programmes in empowering patients for self-management, as well as more optimally make use of scarce human resources [29]. These programmes have relied extensively on trained community health workers and facilitated peer support groups to assist HIV patients in developing the knowledge, skills and confidence to manage their health and interact productively with the health services. This may well be a vital component in an expanded model of NCD care in SA which seeks to increase the levels of patient activation and competency for self-management.

### Limitations

This study had a number of limitations. More women than men agreed to be interviewed, thus limiting the possibility of gaining insight into possible gender differences in the experience of care or self-management. Recall bias was inevitable as patients recalled experiences extending as far back as a few years. Finally, a minority (3) of the interviews could not be conducted in English. In these cases, the researcher, who was not fluent in Xhosa or Afrikaans, required the assistance of an interpreter, which may have limited her ability to build rapport with these patients.

## Conclusion

This study makes a contribution to understanding what NCD patients need from their healthcare providers for effective self-management. Its findings may be particularly pertinent at this time when current models of NCD care are under critical review in SA. Whilst behaviour change is difficult and individual choices are significantly influenced by broader social, cultural and environmental factors, there is much evidence that shows that healthcare providers can play a vital role in motivating and assisting patients in modifying NCD risk behaviours [26, 27]. Policy frameworks, training programmes, resources and tools are needed in South Africa in order to enable healthcare providers to better undertake these tasks. Furthermore, research is required to investigate how proposed models of chronic care, which support NCD patients’ needs for relatedness, autonomy and competence can be integrated into a primary health care system still geared to providing acute, episodic care.

## Footnotes

1 Xhosa is the southernmost branch of the Nguni group of African language, mainly spoken in the Eastern and Western Cape provinces of SA.

2 Afrikaans is a creole language unique to SA and spoken by people of Dutch settler descent and of mixed ancestry.

3 Xhosa is the southernmost branch of the Nguni group of African language, mainly spoken in the Eastern and Western Cape provinces of SA.

4 Afrikaans is a creole language unique to SA and spoken by people of Dutch settler descent and of mixed ancestry.
